# Polish version of SARC-F to assess sarcopenia in older adults: An examination of reliability and validity

**DOI:** 10.1371/journal.pone.0244001

**Published:** 2020-12-21

**Authors:** Roma Krzymińska-Siemaszko, Ewa Deskur-Śmielecka, Aleksandra Kaluźniak-Szymanowska, Arkadiusz Styszyński, Katarzyna Wieczorowska-Tobis

**Affiliations:** Department of Palliative Medicine, Poznan University of Medical Sciences, Poznan, Poland; University of Houston, UNITED STATES

## Abstract

**Introduction:**

SARC-F is a quick questionnaire recommended as a screening tool for sarcopenia. The aim of the study was to translate, adapt, and validate the Polish version of the SARC-F for community-dwelling older adults in Poland.

**Materials and methods:**

We included 160 Polish volunteers aged ≥ 60 years (44% of men). The Polish version of SARC-F was adapted following standardized forward-backward translation procedure. SARC-F was validated against the six sets of diagnostic criteria as the reference standards [developed independently by European Working Group on Sarcopenia in Older People1 (EWGSOP1), European Working Group on Sarcopenia in Older People2 (EWGSOP2), Foundation for the National Institutes of Health (FNIH) Sarcopenia Project, Asia Working Group for Sarcopenia (AWGS), the International Working Group for Sarcopenia (IWGS), and Society on Sarcopenia, Cachexia and Wasting Disorders (SCWD)].

**Results:**

SARC-F score ≥ 4 points was observed in 18.8% of the study population. Cronbach’s alpha was 0.70. The sensitivity of SARC-F varied from 33.3% to 50.0% depending on the diagnostics criteria used, while the specificity was about 85%. Positive predictive value (PPV) was low (about 30%) for five out of six sets of the diagnostic criteria used (EWGSOP2, IWGS, AWGS, FNIH, and SCWD), while the negative predictive value (NPV) was generally high (>88%). The area under the ROC curves (AUC) was 0.652–0.728. SARC-F had the largest AUC against FNIH criteria (0.728), indicating a moderate diagnostic accuracy. Similar results were found for EWGSOP2 and IWGS criteria. The AUC values were below 0.7 for AWGS, SCWD, and EWGSOP1 criteria.

**Conclusion:**

Based on the results, the Polish version of SARC-F shows excellent reliability and good internal consistency. High specificity and high NPV make SARC-F a useful tool to rule-out sarcopenia with high accuracy in community-dwelling older adults, independently of the diagnostic criteria used.

## Introduction

Sarcopenia, a progressive and generalized loss of muscle strength, function and mass, is a severe condition threatening healthy aging [1]. In 2013, Malmstrom and Morley developed the SARC-F questionnaire, composed of 5 simple questions, which is currently regarded as one of the best screening tools for sarcopenia in clinical practice [2]. Other tools, such as the Ishii index [3] and Mini Sarcopenia Risk Assessment Questionnaire (MSRA) [4], are far less commonly used. In September 2018, the Extended European Working Group on Sarcopenia in Older People (EWGSOP2) incorporated the SARC-F questionnaire to updated diagnostic criteria as a screening tool to find cases with the suspicion of sarcopenia [1].

Diagnostics of sarcopenia is complicated, as the assessment of muscle mass and function requires specialized equipment and is time-consuming. Such measurements are an inherent part of all currently available sets of diagnostic criteria for sarcopenia, including European Working Group on Sarcopenia in Older People (EWGSOP1) [5], the Extended European Working Group on Sarcopenia in Older People (EWGSOP2) [1], the Foundation for the National Institutes of Health (FNIH) Sarcopenia Project [6], the Asia Working Group for Sarcopenia (AWGS) [7], the International Working Group for Sarcopenia (IWGS) [8], and the Society on Sarcopenia, Cachexia and Wasting Disorders (SCWD) [9]. Limited access to specialized equipment necessary for muscle mass and function measurement prevents widespread diagnostics of sarcopenia in clinical practice in many countries. Diagnostic of sarcopenia may be also neglected by health care professionals, which is often explained by shortage of time in busy clinical practices. Another reason can be lack of conviction that sarcopenia is a serious condition, and lack of knowledge of treatment methods. The situation is further complicated by several existing definitions and diagnostic criteria of sarcopenia, without a worldwide consensus. Nevertheless, diagnosis of sarcopenia is especially important in several populations. In older people, it is associated with important complications, such as the increased risk of falls and injury, physical disability, dependence, and death [1, 10]. In patients with cancer, it is a prognostic factor of survival [11, 12]. Therefore, there is a considerable need for a short and simple tool suitable for diagnosis of sarcopenia in clinical practice.

To extend the SARC-F questionnaire’s availability, Special Interest Group on Sarcopenia of European Union Geriatric Medicine Society (EuGMS) included language translation and validation of SARC-F among their priorities in 2017 [13]. Such analyses have been conducted by several research teams in the world [14–26]. Following the encouragement of EuGMS, we decided to translate the SARC-F into Polish and validate it against six sets of diagnostic criteria for sarcopenia: EWGSOP1 [5], EWGSOP2 [1], FNIH [6], AWGS [7], IWGS [8] and SCWD [9]. To the best of our knowledge, this is the first validation study employing all currently available sets of diagnostics criteria of sarcopenia in community-dwelling older adults. In 2017 Beaudart et al. [14] assessed the performance of SARC-F against five sets of international diagnostics criteria of sarcopenia available at that time. In addition to the five sets of criteria used in their study, we incorporated recommendations of EWGSOP2, released in September 2018 [1].

## Materials and methods

### Study group

The reliability and validity of the SARC-F questionnaire were assessed in a cross-sectional study conducted between March 2019 and February 2020 in community-dwelling volunteers living in a city of Poznan, one of the largest cities in Poland. The inclusion criteria were age (60 years and older), preserved cognitive function [defined as Abbreviated Mental Test Score (AMTS) ≥ 7 points], ability to maintain standing position (necessary for assessment of body height and composition and Appendicular Lean Mass (ALM) estimation), and the ability to walk a 4-meter distance (to assess the gait speed). Subjects in whom the analysis of body composition with the bioimpedance method (BIA) was impossible because of implanted artificial pacemaker, metal implants, or oedemas were excluded. One hundred seventy persons volunteered for the study. Ten of them were excluded for the following reasons: cognitive impairment (n = 5), having a pacemaker (n = 2), physical disability preventing a 4-m usual walking speed test (n = 3).

Each subject gave written informed consent before they participated in the study. The study was conducted in accordance with the Declaration of Helsinki, and the protocol was approved by the Poznan University of Medical Sciences, Poland (approval No: 872/18).

### Data collection

Face-to-face interviews were conducted to collect sociodemographic (age, sex, marital status, education level, place of residence) and clinical (self-reported comorbidity and number of drugs taken regularly) data. Anthropometric measurements were taken (body mass and height, arm and calf circumference). Nutritional status was screened with Mini Nutritional Assessment (full MNA), independence in basic activities of daily living (ADL) was assessed with the Katz scale, and independence in instrumental activities of daily living (IADL) with the Lawton Scale. The cognitive performance was assessed with the Abbreviated Mental Test Score (AMTS). All the tools used in the study have been described in detail in our previous publication [27].

### Translation and adaptation of the SARC-F questionnaire

The SARC-F questionnaire comprises five domains: 1) strength, 2) assistance in walking, 3) rising from a chair, 4) climbing stairs, and 5) falls. Each domain is scored 0–2, and total score ≥ 4 indicates the risk of sarcopenia [2].

The Polish version of SARC-F was worked out following standardized forward-backward translation procedure [28]. Two Polish native speakers, a geriatrician, and a professional English translator independently translated the original version of SARC-F. The discrepancies between the two translations were discussed in a multidisciplinary team consisting of an English language translator, physiotherapist, a dietitian, and two geriatricians. Since the metric system is used in Poland, the first question of the SARC-F questionnaire ‘How much difficulty do you have in lifting and carrying 10 pounds?' was modified to: ‘How much difficulty do you have in lifting and carrying ‘about 5 kilograms?'. An English native speaker fluent in Polish and without knowledge of the original version of SARC-F performed the back translation. The multidisciplinary team compared the Polish version of the SARC-F and its English back translation and found no disagreement. The final Polish version of SARC-F was completed by ten elderly persons (5 men and 5 women) without cognitive impairment and acute comorbidity to reveal their opinions regarding the questionnaire’s comprehension and cultural relevance. Following Special Interest Group on Sarcopenia EuGMS recommendations, two independent researchers used the Polish version of SARC-F questionnaire in a group of 20 elderly persons (10 men and 10 women) to assess inter-rater reliability and test-retest reliability with a time interval of at least 2 weeks (but no more than 4 weeks) [13]. The Polish version of the SARC-F can be found as S1 Table.

### Assessment of sarcopenia using different criteria

Results of the SARC-F questionnaire were compared with six sets of international diagnostic criteria listed below as the reference standard of sarcopenia diagnosis: (1) the EWGSOP1 [5]; (2) the EWGSOP2 [1], (3) the Foundation for the National Institutes of Health (FNIH) Sarcopenia Project [6], (4) the Asia Working Group for Sarcopenia (AWGS) [7], (5) the International Working Group for Sarcopenia (IWGS) [8], and (6) the Society on Sarcopenia, Cachexia and Wasting Disorders (SCWD) [9].

A detailed description of the parameters used by the international diagnostics criteria is shown in Table 1.

**Table 1:** Six sets of international diagnostic criteria of sarcopenia.

	Low muscle strength	Low muscle mass	Low physical performance	Diagnostic criteria
Sarcopenia according to EWGSOP1	GS < 30 kg for M	ALM/height^2^ ≤ 7.40 kg/m^2^ for M*	UGS ≤ 0.8 m/s for both sexes	UGS + LMM and/or GS + LMM
	GS < 20 kg for W	ALM/height^2^ ≤ 5.60 kg/m^2^ for W*		
Sarcopenia according to EWGSOP2	GS < 27 kg for M		─	GS and/or CST + LMM
	GS < 16 kg for W	ALM/height^2^ ≤ 7.00 kg/m^2^ for M		
	and/or CST > 15 s for five rises	ALM/height^2^ ≤ 5.50 kg/m^2^ for W		
Sarcopenia according to FNIH	GS < 26 kg for M	ALM/BMI < 0.798 for M	UGS ≤ 0.8 m/s for both sexes	GS + LMM + UGS
	GS < 16 kg for W	ALM/BMI < 0.512 for W		
Sarcopenia according to AWGS	GS < 26 kg for M	ALM/height^2^ < 7.00 kg/m^2^ for M	UGS ≤ 0.8 m/s for both sexes	GS + LMM and/or UGS + LMM
	GS < 18 kg for W	ALM/height^2^ < 5.40 kg/m^2^ for W		
Sarcopenia according to IWGS	─	ALM/height^2^ ≤ 7.23 kg/m^2^ for M	UGS < 1.0 m/s for both sexes	LMM + UGS
		ALM/height^2^ ≤ 5.67 kg/m^2^ for W		
Sarcopenia according to SCWD	─	ALM/height^2^ ≤ 7.29 kg/m^2^ for M**	UGS ≤ 1.0 m/s for both sexes	LMM + UGS
		ALM/height^2^ ≤ 5.52 kg/m^2^ for W**		

Notes: M, men; W, women; GS, grip strength; CST, chair stand test; ALM, appendicular skeletal muscle mass; LMM, low muscle mass; BMI, body mass index; UGS, 4m usual gait speed;

* Polish cut-off points for reference population aged 18–40 yrs [38],

** Polish cut-off points for reference population aged 20–30 yrs [39], EWGSOP1, the European Working Group on Sarcopenia in Older People; EWGSOP2, extended group for the European Working Group on Sarcopenia in Older People; FNIH, Foundation for the National Institutes of Health; AWGS, Asian Working Group for Sarcopenia; IWGS, International Working Group on Sarcopenia; SCWD, Society on Sarcopenia Cachexia and Wasting Disorders.

### Measurements of muscle mass, muscle strength, and physical performance

#### Assessment of muscle mass

The muscle mass was measured with the BIA method (InBody 120, Biospace, Seoul, South Korea). The Appendicular Lean Mass (ALM) index [defined as the sum of lean mass of upper and lower limbs (kg) divided by the squared height (m^2^)] was calculated in all subjects. Height was measured with a mobile stadiometer (Tanita, Poznan, Poland). The cut-off points used for the ALM index are shown in Table 1.

#### Assessment of muscle strength

Muscle strength was measured with a handgrip dynamometer (Saehan, Changwon, South Korea) and using The Chair Stand Test (CST). The handgrip strength test was performed in a sitting position, with arms bent to 90 degrees in the elbow and shoulder joint. Measurements for the left and right arms were repeated twice, and the mean value was calculated to give the final score. The results were expressed in kilograms (kg). According to the EWGSOP2 algorithm [1], we additionally used the CST to assess lower limb strength. The time necessary to 5 stands up from a seated position with arms folded across the chest was measured (in seconds, s). The cut-off points for muscle strength parameters are shown in Table 1.

#### Assessment of physical performance

The 4-m usual gait speed test (UGS) was used to assess physical performance. Subjects were allowed to use walking aids (canes, walkers) during the test, if necessary. The time necessary to complete the test was measured with a stopwatch. The results were expressed in meters per second. The cut-off points for the UGS test are presented in Table 1.

### SARC-F reliability and validity

SARC-F was evaluated used the following procedures: (1) the reliability was obtained by the Cronbach alpha and the correlation of each item in the questionnaire with the scale’s total score; (2) the temporal consistency was evaluated by test-retest (2–4 week interval between the two measurements); (3) the criterion validity was assessed through the calculation of the sensitivity, specificity, and positive and negative predictive values of the SARC-F concerning sarcopenia defined according to the EWGSOP1, EWGSOP2, AWGS, IWGS, FNIH, and SCWD criteria; and (4) the associations between SARC-F and physical measurements, such as handgrip strength, CST, UGS, ALM, ALM/BMI, ALM index, and BMI. The SARC-F scale was also validated against other measurements related to sarcopenia, such as (1) age, (2) the Katz Scale to assess basic activities of daily living (ADL), (3) the Lawton Scale to assess instrumental activities of daily living (IADL), (4) the Mini Nutritional Assessment (MNA) to measure the risk of malnutrition. The total score obtained in all the scales was considered.

### Statistical analysis

Statistical analysis was performed using the STATISTICA 12.0 package (StatSoft, Poland). Continuous data were presented as mean ± SD and compared using the Student’s t-test or the Cochran–Cox test, or Mann–Whitney test as appropriate. Categorical variables were expressed as numbers (percentage) and compared with the χ2 test with the Yates correction if applicable. Men and women results were analyzed separately. Reliability was assessed by internal consistency, inter-rater, and test-retest analyses. Internal consistency was tested using Cronbach’s alpha for all five items. Cronbach alpha value ≥0.70, indicating an acceptable level of internal consistency [29]. The test-retest and the inter-rater reliability was evaluated by intraclass correlation coefficient (ICC). The level of agreement was defined as follows: poor <0.5, moderate 0.50–0.75, good 0.75–0.90, and excellent >0.90 [30]. The Spearman test was used to correlate measurements without normal distribution: (1) each item in the SARC-F with the total scale’s score (internal consistency), (2) other measurements related to sarcopenia and the SARC-F total score (validation against other measurements), (3) and other measurements related to sarcopenia and each domain in the SARC-F scale. The difference between the frequencies of sarcopenia obtained by the SARC-F [2], EWGSOP1 [5], EWGSOP2 [1], FNIH [6], AWGS [7], IWGS [8], and SCWD [9] were compared using the χ2.

ROC analyses for the SARC-F based on the six sets of international diagnostic criteria [i.e., reference standard: EWGSOP1 [5], EWGSOP2 [1], FNIH [6], AWGS [7], IWGS [8], and SCWD [9] criteria] were used to analyze sensitivity, specificity, positive and negative predictive values (PPV and NPV), accuracy and AUC (area under the curve). The AUC values >0.9, 0.7 to 0.9, and 0.5 to 0.7 corresponded to the high, moderate, and low diagnostic accuracy of the screening test, respectively [31, 32]. The sensitivity is the proportion of subjects presenting with sarcopenia (based on the reference standard) having been correctly identified as sarcopenic using the screening test (i.e., positive screening test). The specificity represents the proportion of individuals who do not have sarcopenia (based on the reference standard), which were correctly identified as non-sarcopenic using the screening test (i.e., negative screening test). The PPV is a measure of the probability of presenting sarcopenia in case of a positive screening test; in turn, the NPV represents the probability of not having sarcopenia in case of a negative screening test. The accuracy measures the proportion of correct classifications over the total number of classifications. P<0.05 was considered statistically significant.

## Results

### Characteristics of the study group

One hundred sixty persons aged ≥ 60 years (60 to 93 years; 44% of men) were included in the study. Clinical characteristics of the total study population and classified by sex are presented in Table 2.

**Table 2:** Characteristics of the whole study population and according to gender.

Characteristics	Total (n = 160)	Men (n = 71)	Women (n = 89)	p
Age (years) ^a^	72.6 (7.2)	71.6 (7.6)	73.5 (6.7)	0.0795
Age cohort ^b^				
65–74 yrs	101 (63.1)	48 (67.6)	53 (59.6)	0.2408
75 yrs or more	59 (36.9)	23 (32.4)	36 (40.4)	
Level of education ^b^^,^^&^				
no education or primary	7 (4.4)	1 (1.4)	6 (6.9)	0.2009
higher than primary	151 (95.6)	70 (98.6)	81 (93.1)	
Living conditions ^b^^,^^&^				
Living alone	50 (31.6)	11 (15.5)	39 (44.8)	0.0001
Living with others	108 (68.4)	60 (84.5)	48 (55.2)	
Marital status ^b^^,^^&^				
Unmarried	67 (42.4)	18 (25.4)	49 (56.3)	0.0001
Married	91 (57.6)	53 (74.6)	38 (43.7)	
Height (cm) ^a^	163.6 (9.8)	172.1 (6.4)	156.8 (6.0)	0.0000
Weight (kg) ^a^	72.4 (15.7)	79.5 (14.0)	66.8 (14.7)	0.0000
BMI (kg/m^2^) ^a^	27.0 (5.4)	26.8 (4.4)	27.2 (6.1)	0.6830
Low BMI ^b^				
Yes	21 (13.1)	7 (9.9)	14 (15.7)	0.2745
No	139 (86.9)	64 (90.1)	75 (84.3)	
MNA score ^a^	24.9 (3.5)	25.1 (3.1)	24.7 (3.8)	0.8715
MNA status ^b^				
Malnutrition	6 (3.8)	1 (1.4)	5 (5.6)	0.3402
Risk of malnutrition	44 (27.5)	20 (28.2)	24 (27.0)	
Normal nutritional status	110 (68.8)	50 (70.4)	60 (67.4)	
ADL score ^a^	5.8 (0.4)	5.8 (0.5)	5.7 (0.4)	0.0215
ADL, status ^b^				
Independent	158 (98.8)	70 (98.6)	88 (98.9)	0.5789
Partially dependent	2 (1.3)	1 (1.4)	1 (1.1)	
Dependent	0 (0.0)	0 (0.0)	0 (0.0)	
IADL score ^a^	25.5 (2.5)	25.3 (2.5)	25.7 (2.4)	0.2451
AMTS score ^a^	9.4 (0.6)	9.4 (0.7)	9.3 (0.6)	0.0578
Number of regular drugs ^a^	5.9 (3.9)	6.3 (4.0)	5.6 (3.9)	0.2651
Number of chronic diseases ^a^	3.3 (1.8)	2.8 (1.3)	3.7 (2.0)	0.0007
Handgrip strength ^a^	25.2 (9.7)	32.7 (9.1)	19.2 (4.9)	0.0000
Gait speed ^a^	1.0 (0.3)	1.0 (0.3)	0.9 (0.3)	0.0886
Chair stand test (s) ^a^**	12.8 (4.6)	12.9 (4.8)	12.8 (4.5)	0.9769
ALM (kg) ^a^	19.3 (5.0)	23.5 (3.6)	15.8 (2.9)	0.0000
ALM index (kg/m^2^) ^a^	7.1 (1.2)	7.9 (1.0)	6.4 (1.0)	0.0000
Calf circumference ^a^	35.7 (3.8)	36.2 (3.4)	35.3 (4.0)	0.1043
SARC-F score ^a^	1.8 (2.0)	1.4 (1.9)	2.1 (2.0)	0.0107

Notes:

^a^ Data are presented as mean (standard deviation);

^b^Data are presented as n (%);

^&^ data missing for two subjects;

* low BMI, i.e. < 20 if < 70 years, or < 22 if ≥ 70 years [33];

** n = 154, excluding six women who were unable to complete the CST due to low back pain.

Abbreviations: BMI, body mass index; MNA, Mini Nutritional Assessment; ADL, activities of daily living; IADL, instrumental activities of daily living; AMTS, Abbreviated Mental Test Score; ALM, appendicular lean mass.

More than one-third of the study participants was ≥ 75 yrs. Women were older than men (73.5±6.7 vs. 71.6±7.6 yrs, p = 0.08). Almost half of the participants (women twice as common as men, p<0.001) were unmarried, including widows/widowers (n = 52), those who were divorced or separated from their spouses (n = 7), as well as those never married (n = 8). One-third of the study population was living alone (women 3-times more common than men, p<0.001). The majority of subjects had secondary education or higher.

Women were significantly shorter than men (156.8±6.0 vs. 172.1±6.4 cm) and had lower body mass (66.8±14.7 vs. 79.5±14.0 kg), but their BMI did not differ significantly (27.2±6.1 vs. 26.8±4.4 kg/m^2^). Almost one out of eight persons had low BMI (low BMI, i.e. <20 if under 70 years, or <22 if 70 years or older according to the Global Leadership Initiative on Malnutrition criteria) [33]. The prevalence of low BMI was higher in women. The mean full MNA score indicated normal nutritional status, however in 30% of the study population malnutrition or risk of malnutrition was diagnosed. Almost all participants were independent according to the Katz scale. No differences were found between men and women for the mean ADL and IADL scores. Participants declared having three chronic diseases and taking six medications daily on average. The most commonly self-reported chronic diseases were as follows: hypertension (58.9%), chronic obstructive pulmonary disease (32.3%), cardiovascular disease (24.1%), dyslipidemia (20.3%), diabetes (18.4%), and osteoporosis/osteopenia (13.3%); (data available in 158/160 participants).

Upper limb muscle strength was higher in men than in women (32.7±9.1 vs. 19.2±4.9 kg, respectively; p<0.001). Lower limb strength assessed with the Chair Stand Test was similar in both sexes. However, the 4-m usual gait speed tended to be lower in men (0.9±0.3 vs. 1.0±0.3 m/s, respectively; p = 0.09). Women had lower ALM (15.8±2.9 vs. 23.5±3.6 kg; p<0.001) and ALM Index (6.4±1.0 vs 7.9±1.0 kg/m^2^; p<0.001). The SARC-F scores were higher in women as compared to men (2.1±2.0 vs. 1.4±1.9 points, respectively; p<0.05).

### Prevalence of sarcopenia

Table 3 shows the frequency of sarcopenia in the total study and both sexes. The SARC-F score was equal to or higher than 4 in nearly 20% of the study population, indicating the risk of sarcopenia. The risk of sarcopenia was 1.5 times more frequent in women than in men; however, the difference was not significant. The prevalence of sarcopenia varied considerably between the six sets of international criteria used in our study. The lowest percentage was found when EWGSOP2 criteria [1] were used—approximately 11% of the study population, and the highest with EWGSOP1 criteria from 2010 [5]—nearly 21% of participants. This difference can be explained by higher cut-off points for ALM Index and handgrip strength in the EWGSOP1 algorithm, as well as due to inclusion in the updated EWGSOP2 algorithm the assessment of lower limb strength by means of Chair Stand Test. Prevalence of sarcopenia was higher in women when assessed with all diagnostic criteria, except for EWGSOP1; however, the difference was not significant.

**Table 3:** Prevalence of sarcopenia according two different questionnaires and six sets of international diagnostic criteria of sarcopenia.

Characteristics		Total (n = 160)	Men (n = 71)	Women (n = 89)	p
**Questionnaires to assess the risk of sarcopenia**					
SARC-F classification	Risk of Sarcopenia	30 (18.8)	10 (14.1)	20 (22.5)	0.1769
	Without the Risk of Sarcopenia	130 (81.3)	61 (85.9)	69 (77.7)	
**Sets of international diagnostic criteria of sarcopenia**					
EWGSOP1 classification	Sarcopenia	33 (20.6)	17 (23.9)	16 (18.0)	0.3541
	Non-sarcopenia	127 (79.4)	54 (76.1)	73 (82.0)	
EWGSOP2 classification	Sarcopenia*	18 (11.3)	7 (9.9)	11 (12.4)	0.6190
	Non-sarcopenia	142 (88.8)	64 (90.1)	78 (87.6)	
FNIH classification	Sarcopenia	19 (11.9)	6 (8.5)	13 (14.6)	0.2317
	Non-sarcopenia	141 (88.1)	65 (91.5)	76 (85.4)	
AWGS classification	Sarcopenia	23 (14.4)	7 (9.9)	16 (18.0)	0.1459
	Non-sarcopenia	137 (85.6)	64 (90.1)	73 (82.0)	
IWGS classification	Sarcopenia	22 (13.8)	8 (11.3)	14 (15.7)	0.4154
	Non-sarcopenia	138 (86.3)	63 (88.7)	75 (84.3)	
SCWD classification	Sarcopenia	24 (15.0)	9 (12.7)	15 (16.9)	0.4622
	Non-sarcopenia	136 (85.0)	62 (87.3)	74 (83.1)	

Notes: Data are presented as n (%);

*sarcopenia confirmed.

Abbreviations: EWGSOP1, the European Working Group on Sarcopenia in Older People 1; EWGSOP2, extended group for the European Working Group on Sarcopenia in Older People 2; FNIH, the Foundation for the National Institutes of Health; AWGS, Asian Working Group on Sarcopenia; IWGS, the International Working Group on Sarcopenia; SCWD, the Society on Sarcopenia, Cachexia and Wasting Disorders.

### Reliability

Table 4 shows the internal consistency of the SARC-F. Cronbach’s alpha was 0.70. All items were correlated to the scale’s total score (rho ranging from 0.52 to 0.79). There was a statistically significant correlation between the SARC-F total score and other measures related to sarcopenia: age, handgrip strength, Chair Stand Test, 4-m usual gait speed, ALM/BMI, ALM, ALM index, ADL, IADL, and MNA (Table 5). Spearman correlations ranged from -0.51 (for MNA) to 0.42 (for CST). The only exception was the BMI index, for which no significant correlation with SARC-F total score was found.

**Table 4:** Internal consistency of the Polish version of the SARC-F questionnaire.

SARC-F item	Correlation	p-value
Strength	0.785	0.0000
Assistance with walking	0.519	0.0000
Rising from a chair	0.586	0.0000
Climbing Stairs	0.670	0.0000
Falls	0.523	0.0000

Notes: Cronbach’s alpha = 0.70. The item-total score correlations were analyzed by the Spearman test.

**Table 5:** Validation between the SARC-F (each domain and total score) and other related measurement.

	Strength		Assistance in Walking		Rising from a Chair		Climbing Stairs		Falls		Total Score	
	Correlation	p	Correlation	p	Correlation	p	Correlation	p	Correlation	p	Correlation	p
Age	0.108	0.1729	0.158	0.0462	0.280	0.0003	0.065	0.4155	0.172	0.0296	0.229	0.0036
Handgrip strength	-0.466	0.0000	-0.320	0.0000	-0.313	0.0001	-0.226	0.0040	-0.174	0.0281	-0.462	0.0000
Chair Stand Test	0.223	0.0055	0.390	0.0000	0.325	0.0000	0.384	0.0000	0.093	0.2522	0.420	0.0000
Usual gait speed	-0.304	0.0001	-0.427	0.0000	-0.267	0.0006	-0.364	0.0000	-0.231	0.0033	-0.432	0.0000
ALM	-0.320	0.0000	-0.162	0.0412	-0.068	0.3947	-0.011	0.8944	-0.034	0.6661	-0.200	0.0111
ALM index	-0.311	0.0001	-0.154	0.0521	-0.033	0.6813	-0.013	0.8722	-0.026	0.7489	-0.192	0.0150
BMI	-0.209	0.0079	-0.008	0.9196	0.110	0.1665	0.066	0.4099	-0.003	0.9662	-0.053	0.5082
ALM/BMI	-0.197	0.0127	-0.166	0.0354	-0.128	0.1080	-0.065	0.4107	-0.073	0.3607	-0.192	0.0151
ADL	-0.279	0.0004	-0.247	0.0016	-0.266	0.0007	-0.295	0.0002	-0.375	0.0000	-0.413	0.0000
IADL	-0.280	0.0003	-0.404	0.0000	-0.411	0.0000	-0.457	0.0000	-0.248	0.0015	-0.495	0.0000
MNA	-0.403	0.0000	-0.280	0.0003	-0.180	0.0230	-0.430	0.0000	-0.217	0.0059	-0.508	0.0000

Abbreviations: ALM, appendicular lean mass; BMI, body mass index; ADL, activities of daily living; IADL, instrumental activities of daily living; MNA—Mini Nutritional Assessment.

### Inter-rater reliability and test-retest reliability

Twenty volunteers (10 women and 10 men) aged 65–91 years (mean 76.0±7.3 yrs) were independently examined by two researchers (a physician-geriatrician and a physiotherapist) in two different offices. The mean age was 77.4±7.5 yrs in women and 74.6±7.2 yrs in men (p>0.05). Inter-rater reliability was determined by ICC. There was an excellent agreement between the results obtained by the two researchers, as indicated by the ICC value 0.993, and a very strong positive correlation (Rs = 0.995, p<0.001). The same 20 persons were re-examined 2–4 weeks later by one of the researchers (the physiotherapist) for the test-retest reliability check. The results were concordant in 75%. The intraclass correlation coefficient for test-retest was 0.9280 (p<0.001), indicating an excellent agreement.

### Clinical validation of the Polish SARC-F

As shown in Table 6, the sensitivity of SARC-F ranged from 33.3 to 50%. It was the lowest for the EWGSOP1 criteria (based on which sarcopenia was diagnosed in the highest proportion of participants—almost 21%). The highest sensitivity of SARC-F was found for the EWGSOP2 criteria (sarcopenia diagnosed in only 11% of subjects). The specificity of SARC-F was about 85% for all the international sets of diagnostic criteria for sarcopenia used in the study. SARC-F positive predictive value (PPV) was 30% when EWGSOP2, IWGS, AWGS, FNIH, and SCWD criteria were employed, and negative predictive value (NPV) was generally high (85%). The highest PPV and the lowest NPV was found for EWGSOP1 criteria. The accuracy of SARC-F for the total study population and all diagnostic criteria was very alike (74–81%). The AUC values ranged 0.652–0.728. SARC-F had the largest AUC against FNIH criteria (0.728), indicating a moderate diagnostic accuracy. Similar results were found for EWGSOP2 and IWGS criteria. The AUC values were below 0.7 for AWGS, SCWD, and EWGSOP1 criteria.

**Table 6:** Sensitivity, specificity, positive and negative predictive values and receiver operating characteristic curve model of the SARC-F questionnaires against six sets of international diagnostic criteria of sarcopenia.

	Sensitivity (%)			Specificity (%)			PPV (%)			NPV (%)			Accuracy			AUC		
	M	W	Total	M	W	Total	M	W	Total	M	W	Total	M	W	Total	M	W	Total
**EWGSOP1**	29.4	37.5	33.3	90.7	80.8	85.0	50.0	30.0	36.7	80.3	85.5	83.1	76.1	73.0	74.4	0.718	0.616	0.652
**EWGSOP2**	42.9	54.6	50.0	89.1	82.1	85.2	30.0	30.0	30.0	93.4	92.8	93.1	84.5	78.7	81.3	0.699	0.720	0.711
**FNIH**	16.7	61.5	47.4	86.2	84.2	85.1	10.0	40.0	30.0	91.8	92.8	92.3	80.3	80.9	80.6	0.636	0.782	0.728
**AWGS**	42.9	37.5	39.1	89.1	80.8	84.7	30.0	30.0	30.0	93.4	85.5	89.2	84.5	73.0	78.1	0.699	0.635	0.670
**IWGS**	37.5	42.9	40.9	88.9	81.3	84.8	30.0	30.0	30.0	91.8	88.4	90.0	83.1	75.3	78.8	0.764	0.682	0.721
**SCWD**	33.3	40.0	37.5	88.7	81.1	84.6	30.0	30.0	30.0	90.2	87.0	88.5	81.7	74.2	77.5	0.702	0.642	0.672

Abbreviations: M, men; W, women; PPV, positive predictive values; NPV, negative predictive values; AUC, area under the curve; EWGSOP1, the European Working Group on Sarcopenia in Older People; EWGSOP2, extended group for the European Working Group on Sarcopenia in Older People; FNIH, the Foundation for the National Institutes of Health; AWGS, Asian Working Group on Sarcopenia; IWGS, the International Working Group on Sarcopenia; SCWD, the Society on Sarcopenia, Cachexia and Wasting Disorders

## Discussion

### Reliability and validity

The translated and culturally adapted Polish version of the SARC-F questionnaire showed excellent inter-rater reliability and test-retest reliability, and acceptable internal consistency (the Cronbach’s alpha 0.70). The SARC-F internal consistency was previously assessed in some validation studies; most of them gave results similar to those obtained in our study. For example, Malmstrom et al. found a very high Cronbach’s alpha (0.76–0.81) for the original version of SARC-F [34]. In 2019, Sanchez-Rodriguez et al. published similar results (Cronbach’s alpha 0.779) for the Spanish version of SARC-F [16]. In contrast, insufficient internal consistency (Cronbach’s alpha below the threshold 0.65) was found for the Japanese version of the SARC-F and the version translated into Spanish and culturally adapted for the Mexican population (Cronbach’s alpha 0.610 and 0.641, respectively [19, 20].

In line with the results of some previous studies, we have demonstrated a correlation between SARC-F and some parameters associated with sarcopenia, such as age, low gait speed, low upper and lower limb strength, and low ALM [19, 20, 24]. Similarly to the findings of Kera et al. [19], we have observed a correlation between SARC-F results and the ability to perform basic and instrumental activities of daily living (assessed with Katz and Lawton Scales). Based on our analysis, the Polish version of the SARC-F showed adequate validity as a diagnostic tool for sarcopenia.

### Clinical validation

The results of clinical validation of the Polish version of SARC-F against six sets of international diagnostic criteria for sarcopenia showed low sensitivity (men: 16.7–42.9%; women: 37.5–61.7%), but high specificity (men: 88.7–91.2%; women: 80.8–84.2%) and high negative predictive value (men: 80.3–93.4%; women: 85.5–92.8%). Therefore, the SARC-F questionnaire is a suitable tool for excluding patients without sarcopenia. A negative result of SARC-F makes the diagnosis of sarcopenia less probable, thus eliminating the need for costly diagnostics and the use of specialist equipment (e.g., dual energy X-ray absorptiometry (DEXA) or BIA for muscle mass assessment).

We have analyzed the performance of the Polish version of SARC-F against all currently available sets of international diagnostic criteria for sarcopenia. A similar approach was adopted in some earlier SARC-F validation studies, assessing the psychometric properties of this questionnaire against at least three diagnostic algorithms [14, 15, 18, 20, 21, 25]. Another approach used in validation studies consists in the use of only one or two diagnostic criteria, the most popular being EWGSOP1 and EWGSOP2 [16, 17, 19, 22–24, 26]. For example, the Spanish version of SARC-F [16], validated in a group of 90 community-dwelling adults aged 65 and more, showed high sensitivity (approximately 80%), but only moderate specificity (approximately 50%) against the two sets of diagnostic criteria used in the analysis (EWGSOP1 and EWGSOP2). Similar specificity (47–54%) was demonstrated for the German translation of SARC-F in a validation study involving 117 older persons (mean age 79.1±5.2 years) and using EWGSOP1 and EWGSOP2 criteria as a reference standard [17]. The sensitivity of the German version was lower than the Spanish one (50–63%). The results of both German and Spanish studies are divergent to our analysis, which demonstrated much higher specificity (85% against EWGSOP1 and EWGSOP2 criteria) but lower sensitivity (33.3% against EWGSOP1 and 50.0% against EWGSOP2 criteria). We have searched the Medline database for validation studies of language versions of the SARC-F questionnaire. We have found 13 studies, most of which performed in community-living older adults [14–26]. When EWGSOP1 criteria were used as a diagnostic standard, the authors reported on low sensitivity and high specificity of SARC-F. For example, the sensitivity of Turkish version of SARC-F was 25%, and the specificity 81% [15]. The sensitivity of the Korean version of SARC-F was very similar to the Turkish one (25.3%), but its specificity was even higher (>91%) [21]. In the analysis performed by Parra-Rodriguez for the Spanish translation culturally adapted for the Mexican population, the sensitivity of SARC-F was 35%, and the specificity 82% [20]. All these results are in line with our findings for the Polish translation of SARC-F.

To the best of our knowledge, the lowest sensitivity of SARC-F against EWGSOP1 diagnostic criteria was found in a validation study conducted by Woo et al. [18] in a population of 4000 community-living Chinese people aged 65 years and older. The sensitivity was only 4.2% in men and 9.9% in women. Similarly, low sensitivity (8%) was documented in a group of 734 older community-dwelling Japanese people in a study performed by Kera et al. [19]. The authors of both studies reported an excellent specificity of the SARC-F questionnaire (>94%). A meta-analysis assessing the screening ability of SARC-F, which included six studies published before September 2017, showed low sensitivity (14–21%) and high specificity (90–93%) of this tool against EWGSOP1 as well as IWGS and AWGS criteria [35]. It should be emphasized that higher sensitivity of SARC-F has been found in some more recent validation studies, published since 2018, and mentioned before [16, 17, 22]. Surprisingly high values of SARC-F sensitivity and specificity against EWGSOP2 criteria (92.9% and 98.1%, respectively) were published by Zasadzka et al. [22] in April 2020. The study analyzed the psychometric properties of the Polish version of SARC-F using the EWGSOP2 diagnostic criteria as the only reference standard. The study of Zasadzka et al. was performed in the same geographical region of Poland (Grand Poland district) as our analysis. The BIA method was used to assess muscle mass in both studies. Similarly to our results, the percentage of subjects with SARC-F scores indicative of sarcopenia was about 20%. SARC-F positive older adults had similar age (75.6±8.8 yrs in our study vs. 75.0±5.1 yrs in [22]) and SARC-F total score (5.2±1.5 vs. 5.0±1.5 points, respectively) in both studies. In contrast, our study population was twice as numerous (160 vs. 67, respectively), and the percentage of men was doubled in our study (44% vs. 19%). Despite numerous similarities, we observed much lower values for SARC-F sensitivity (50.0%) and specificity (85.0%). This discrepancy may be attributed to the lower percentage of subjects with sarcopenia based on EWGSOP2 criteria in our study (11% vs. 21%). Other factors that could have contributed to this difference, such as comorbidities and number of drugs, have not been analyzed in the study of Zasadzka et al. [22]. In line with our findings, most international validation studies reported on low sensitivity and high specificity of SARC-F [15, 18–21, 23, 26, 35].

One limitation of our study is the use of the BIA method for muscle mass assessment instead of more precise magnetic resonance imaging (MRI), computed tomography (CT) or DEXA. While the evaluation of muscle mass based on whole body MRI is regarded as the gold standard, rarely it is used in clinical practice and research studies involving larger populations due to its high cost [36]. Regional measurements of muscle size by MRI or CT performed for other reasons are also used to assess muscle properties; however, they were unavailable for this research. DEXA is often used in clinical studies, but its application is restricted to two body scans per year, which limits the possibilities of regular assessment of body mass (e.g. before and after a therapeutic intervention) [37]. MRI, CT and DEXA alike are costly methods, requiring specialized staff to operate them. BIA method is much simpler and non-invasive, thus the assessment can be repeated without restrictions. The equipment is portable, which makes it possible to measure the muscle mass at the patient’s home. The BIA method is recommended by some international groups, such as EWGSOP1 [5], EWGSOP2 [1], AWGS [7], as a cheaper alternative option for muscle measurement. It has been reported, however, that BIA results may differ depending on the hardware and method of validation of the algorithm [36].

A strong point of our SARC-F validation analysis is that we were the first to use all currently available sets of international diagnostic criteria for sarcopenia as a reference standard [there are six of them, developed independently by European Working Group on Sarcopenia in Older People 1 (EWGSOP1) [5], European Working Group on Sarcopenia in Older People 2 (EWGSOP2) [1], Foundation for the National Institutes of Health (FNIH) Sarcopenia Project [6], Asia Working Group for Sarcopenia (AWGS) [7], the International Working Group for Sarcopenia (IWGS) [8], and Society on Sarcopenia, Cachexia and Wasting Disorders (SCWD) [9].

## Conclusions

The Polish version of the SARC-F questionnaire shows excellent reliability and good internal consistency. High specificity and high negative predictive value make SARC-F a useful tool to rule-out sarcopenia with high accuracy in community-dwelling older adults, independently of the diagnostic criteria used. Screening with the SARC-F questionnaire will enable reducing unnecessary and costly measurements required by all currently available sarcopenia diagnostic criteria sets.

## Supporting information

S1 Table Polish version of SARC-F. (DOCX)
