# Supplementary material for: Polish version of SARC-F to assess sarcopenia in older adults: An examination of reliability and validity
Source: PLoS One. 2020 Dec 21;15(12):e0244001. doi: 10.1371/journal.pone.0244001 (PMC7751882; doi:10.1371/journal.pone.0244001)
Supplement: S1 Table — (DOCX) [file pone.0244001.s001.docx]

**S1 Table. Polish Version of SARC-F**

| **SARC-F: PROSTY KWESTIONARIUSZ DO SZYBKIEJ DIAGNOZY SARKOPENII** | | |
| --- | --- | --- |
| **Elementy oceny** | **Pytanie** | **Punktacja** |
| **Siła** | Ile trudności sprawia Panu/Pani podniesienie i przeniesienie około 5 kg? | Nie sprawia wcale = 0  Sprawia trochę = 1  Sprawia dużo albo nie jest w stanie wykonać = 2 |
| **Pomoc przy chodzeniu** | Ile trudności sprawia Panu/Pani przejście przez pokój? | Nie sprawia wcale = 0  Sprawia trochę = 1  Sprawia dużo albo musi użyć przyrządów pomocniczych (kuli/laski/balkonika/chodzika) albo nie jest w stanie wykonać = 2 |
| **Wstawanie z krzesła** | Ile trudności sprawia Panu/Pani wstawanie z krzesła lub łóżka? | Nie sprawia wcale = 0  Sprawia trochę = 1  Sprawia dużo albo nie jest w stanie wykonać bez pomocy = 2 |
| **Wchodzenie po schodach** | Ile trudności sprawia Panu/Pani pokonanie 10 stopni? | Nie sprawia wcale = 0  Sprawia trochę = 1  Sprawia dużo albo nie jest w stanie wykonać = 2 |
| **Upadki** | Ile razy upadł/a Pan/Pani w ostatnim roku? | Nie upadł/a wcale = 0  Upadł/a 1-3 razy = 1  Upadł/a 4 i więcej razy = 2 |
| **Suma punktów (0-10 punktów)** | |  |
| **Interpretacja wyniku: ≥ 4 punktów - podejrzenie sarkopenii** | | |

**Original Version of the SARC-F**

| **SARC-F: A SIMPLE QUESTIONNAIRE TO RAPIDLY DIAGNOSE SARCOPENIA** | | |
| --- | --- | --- |
| **Component** | **Question** | **Scoring** |
| **Strength** | How much difficulty do you have in lifting and carrying 10 pounds? | None = 0  Some = 1  A lot or unable = 2 |
| **Assistance in walking** | How much difficulty do you have walking across a room? | None = 0  Some = 1  A lot, use aids, or unable = 2 |
| **Rise from a chair** | How much difficulty do you have transferring from a chair or bed? | None = 0  Some =1  A lot or unable without help =2 |
| **Climb stairs** | How much difficulty do you have climbing a flight of 10 stairs? | None = 0  Some =1  A lot or unable = 2 |
| **Falls** | How many times you fallen in the past year? | None =0  1-3 falls = 1  4 or more falls = 2 |
| **sum of points (0-10 points)** | |  |
| **Interpretation of the result: ≥ 4 points – probable sarcopenia** | | |

**From Malmstrom TK, Morley JE. SARC-F: a simple questionnaire to rapidly diagnose sarcopenia. J Am Med Dir Assoc. 2013;14(8):531-532.**
